# Immigrant pentecostalism in the emergence of the COVID-19 crisis: reactions and responses from the Universal Church of the Kingdom of God in Berlin

**DOI:** 10.1007/s41682-022-00141-0

**Published:** 2022-12-15

**Authors:** Stefan van der Hoek

**Affiliations:** Research Center for Religion and Education, Faculty of Theology, Friedrich-Schiller-University, Fürstengraben 6, 07743 Jena, Germany

**Keywords:** COVID-19, Universal Church of the Kingdom of God, Pentecostalism, Germany, Third Space, Lusospheres

## Abstract

This article seeks to analyze the reactions and responses of the *Universal Church of the Kingdom of God* (UCKG) in Berlin to the global COVID-19 crisis. Although the UCKG has been the subject of multiple international types of research regarding the spread of the COVID-19 virus, this article shows that the UCKG’s global network of local churches must be differentiated in their responses and reactions to the global pandemic. This article traces and analyzes how the local UCKG in Berlin responded to the pandemic in its respective conditions and differed from the global network in the emergence of a pandemic in its rhetoric and discourse, using the concept of the *Third Space*. For this purpose, the services and sermons of local pastors were recorded to analyze how the discourse toward the COVID-19 crisis changed during the period of occurrence and awareness of a global pandemic. The results show how the church has adapted to the local restriction and regulations and reflect the international literature on how the UCKG’s mother church in Brazil acted in comparison. The church and its pastors in Germany responded to the global pandemic in three primary ways: they assigned to authorities’ guidelines, provided sermons with undertones of spiritual warfare, and rejected the interpretation of interdependencies between demons and health issues.

## Introduction

The rapid spread of the COVID-19 virus since its discovery and medical classification in 2019 has caused massive effects and reactions in all spheres of social life, including the religious aspect. Religious organizations and communities have been challenged in particular by the sudden emergence of the COVID-19 crisis and its accompanying laws and regulations. State authorities were initially irritated about whether to curtail the basic rights of freedom of assembly or respond to the pandemic proportionately and flexibly. Churches and state authorities were requested to change and to re-negotiate established structures, rules, traditions, and practices.

Germany was hit comparatively late by the COVID-19 pandemic in the spring of 2020. The media disseminated the confrontation with the hazards and risks of a new unknown virus. Preparations for containment were made in advance, and media coverage from the events of other countries such as China, Italy, and Spain made a significant contribution to sensitizing the population and government in Germany. The very first infection in Germany, at the end of January 2020, coincided with a strong concentration of religious festivals. The Jewish festivals of Purim, Passover, and Shavuot took place between February and May 2020. Christian festivals, such as the rather Catholic Carnival followed by both Catholic and Protestant Holy Week, Good Friday, Easter, and Pentecost took place in this period as well. Also, on the Muslim side, the month of Ramadan and its festivals also coincided with the time of the arrival of COVID-19. Given this concentration of religious festivals, protests against the curtailment of assembly freedoms and freedoms of worship were comparatively quiet among religious actors in Germany. Social distancing has been recognized by most churches, synagogues, and mosques as an effective measure in containing the virus and religious organizations have therefore made an acknowledged contribution to civil society (Yendell et al. 2021, p. 96). However, the protests and demonstrations against the new regulations and the lockdown were also accompanied by religious actors but fed much more by groups without any specific religious background (Nachtwey et al. 2020, p. 37).

In the global context of the pandemic, however, protest against and even denial of the existence of COVID-19 became visible among religious actors (Usarski and Py 2020, p. 165), especially the spectrum of North and South American evangelicalism and Pentecostalism increasingly moved into the focus of public media (Leśniczak 2022, p. 182). In particular, those who preached divine protection for the nation and their own religious community within the framework of so-called *Prosperity Theology* were addressed and problematized (Levin 2020, p. 2219).

The rapid spread of the COVID-19 virus in Brazil and the high number of deaths associated with COVID-19 infection led to the assumption that the Brazilian government’s loose contact restrictions and general downplaying of the virus’ dangerousness by President Bolsonaro in combination with the discourses of religious leaders of Pentecostal churches, might have played indeed a significant role (Yendell et al. 2021, p. 51). In this context, the Brazilian *Universal Church of the Kingdom of God* (UCKG) has been frequently addressed as a protagonist in the critique of measures to contain the COVID-19 virus in Brazil (Capponi 2020; Serrão and Chaves 2020; Yendell et al. 2021; Martins and Miletta 2021; von Sinner and Zeferino 2022).

Given the international attention the Brazilian UCKG on the global spread of the pandemic received and the demand to deal with evangelical and Pentecostal groups in a differentiated way with respect to their heterogeneous nature, this article aims to inquire into the specific contexts within the diverse field of immigrant Pentecostalism by examining the role of the headquarter of the UCKG in Berlin, Germany. The interest of this study is to trace the dynamic process of religious networks on the basis of circulating discourses in the context of the COVID-19 pandemic. Transnational religious communities may differ from the mother churches in that they associate and identify with and are strongly influenced by local conditions, especially when it comes to a global pandemic. With this line of inquiry, this article aims to connect to a turn in religious studies, which calls for an anthropology of Christianity that pays attention to the respective idiosyncrasies and dynamics of individual communities and singular believers and does not preliminarily assume centralism of religious communities (Robbins 2007, p. 6). This can be studied particularly well in the case of transnational religious communities. In order to notice these differences and to sensitize them, conceptual tools are needed that can be taken up by postcolonial studies. Processes of transnationalization refer to the actual spaces of action of religious communities that spread from one country to another. In light of the spread of so-called ‘world religions’, processes of globalization and translocalization are surprising constants in the history and present of religion and the subject of much sociological theory and research on religion. Therefore, in contrast to the thematization of transnational migration processes, I propose here an approach from the perspective of transnational socialization through the concept of *Third Space* originating from postcolonialism. *Third Space* was conceptualized by Homi K. Bhabha and refers to a place that is not primarily determined as a physical location, but as an intermediary sphere (Bhabha 1994, p. 54). Bhabha writes:

“[…] multinational networks as existing somewhere beyond our perceptual, mappable experience, he can only envisage the representation of global ‘difference’ by making a renewed appeal to the mimetic visual faculty—this time in the name of an ‘incommensurability-vision’. What is manifestly new about this version of international space and its social (in)visibility, is its temporal measure […]. The non-synchronous temporality of global and national cultures opens up a cultural space—a third space—where the negotiation of incommensurable differences creates a tension peculiar to borderline existences.” (Bhabha 1994, p. 312)

With a focus on the tensions in which the formation of a borderline existence takes place and where the *Third Space* is created, the following field study results will be examined.

In the sociology of religion, the concept of the *Third Space* in the structure of transnational identity formation describes the place of a new localization, which configures itself in creative dealings with the home and the migration cultures, with the orthodox and the heterodox, with the past and the present, and creates therefore new identities in diaspora situation. The social order inside the transnational religious organization thus finds itself in the paradoxical situation of being permanently questioned by itself and its actors. The pluralization of religious interpretations and worldviews plays an essential role in dealing with contingency in a space of flows. In this sense, this article pursues the approach that contradiction, dissent, and protest are part of modernity, and those religious communities, which are commonly framed as ‘the evangelicals’, ‘the Pentecostals’, or even UCKG can be described more accurate when sociological research on religion conceives them not per se as antagonists of modern societies but as formative co-actors of plural societies, that deserves a closer perspective regarding its paradoxes and arbitrariness in reflection with the *Third Space*. Hence, the concept opens up the possibility of locating new identities that are not described solely from processes of assimilation, hybridization, or syncretism, but detach themselves from cultural attributions.

In somewhat simplified terms, the core hypothesis is that the relationship between different religious communities and the relationship within a transnational religious community can be described as an ambivalent hybridization process of incommensurability.

The current trajectories of migrants from the Global South are perceived as one of the most significant drivers of religious pluralization in Germany (Zurlo et al. 2020, p. 12). Given the theoretical concept of *Third Space*, the empirical part of this article will focus on the local headquarter of the UCKG in Germany and its responses and reactions to the COVID-19 pandemic. At the same time, this article connects to a current migration studies debate initiated under the term “lusospheres” (Oosterbaan et al. 2020), which refers to migration movements and dynamics among Portuguese-speaking communities (Ibid, p. 4). Within the Lusophony world, Brazil has become an international hub for the global mobility of religion in recent decades due to increased emigration, mobility, and (trans-)cultural influence (Rocha and Vásquez 2013; Rocha 2017; Oosterbaan et al. 2020; Oro 2019, p. 309). According to the Brazilian Ministry of Foreign Relations, in 2020 more than 144,000 Brazilian migrants lived in Germany (Ministério das Relações Exteriores 2021, p. 9). The majority of these Brazilian migrants in Germany reside in major cities, such as Frankfurt, Munich, and Berlin (Kürbis 2021, p. 41). Despite the growing number of Brazilian communities in Germany, to date, there has been little research on the religious institutions of Brazilian migrants in Germany. In this article, the Brazilian community will not be understood as an entity, but rather the diversity among religious actors originating from Brazil will be particularly considered.

## The UCKG in Brazil and Abroad

The *Universal Church of the Kingdom of God* (UCKG) is a Brazilian Pentecostal megachurch that has spread worldwide in recent decades and has been the subject of international research on global Pentecostalism (Openshaw 2021; van der Hoek 2021; Vasconcelos de Castro Moreira 2021). The UCKG is a prominent example of so-called ‘Neo-Pentecostalism’ (Freston 1993, p. 95; Chesnut 2003, p. 12; Mattos 2021, p. 401; Fernandes 2022, p. 55). This classification however is an academic designation that does not necessarily correspond to the self-description of the respective churches. Neo-Pentecostalism in Brazil has pushed the numbers of Pentecostal Christians in the national statistics tremendously, by addressing social and spiritual needs and making Brazil the country with the highest Pentecostal population worldwide (Fernandes 2022, p. 2). Following the academic definitions, Neo-Pentecostalism has a particular focus on the prosperity gospel and spiritual warfare (McClymond 2021, p. 452). Others classify Brazilian Neo-Pentecostalism as postmodern and a syncretized Religion with elements of afro-Brazilian Religions and reinterpretations through the lens of television culture (Chesnut 1997, p. 45). These two theological perspectives are essential to the expansion of the churches and their doctrines and practices. They explain the church’s practice of demanding large amounts of money from members while teaching an incessant battle against the devil and demons, who are blamed for financial misfortune as well as physical, psychological, or social problems (de Vasconceles 2022, p. 109). The UCKG is described within Brazilian Neo-Pentecostalism as a church that primarily attracts the most underprivileged classes and is particularly heavily frequented by female adherents compared to the already high rates of feminization in Brazilian Pentecostalism (Chesnut 2003, p. 61). Class distinctions also seem to be reflected in Brazilian Pentecostal churches in Brazil and abroad, as Rocha showed with the example of Brazilian churches in Australia (Rocha 2021, p. 247) or as Koehrsen has pointed out with regard to the diaspora of Brazilian churches in Argentina (Koehrsen 2016, p. 17).

Originally, the UCKG was founded in the late 1970s by former members of the *Nova Vida Pentecostal Church*, which was established by Canadian missionary Robert McAllister (Mattos 2021, 401). The exact origins of the UCKG are somewhat ambiguous. While many attributes the year of foundation to year 1977 (Openshaw 2021, p. 645), others cite 1979 as the founding year of the UCKG (Mattos 2021, p. 401).

The current bishop of the church and one of its founding members is Edir Macedo, who remains its highest and most prominent bishop today. He is among the richest and wealthiest personalities in Brazil, and his private wealth far exceeds that of other prominent Pentecostal televangelists, such as Kenneth Copeland, David Oyedepo, or Pat Robertson. Although his private wealth may well be linked to the expansive donation practices of the UCKG and it should not be ruled out that the pastors and bishops of the UCKG enrich themselves from the donations of the worshippers, Macedo’s immense private fortune, estimated by Forbes at over $1 billion, is based on various sources of income. Macedo successfully expanded his income as a pastor into various business sectors. In the 1980s and 1990s Macedo bought television and radio stations, which he developed into Brazil’s second-largest media group. In addition to banks, real estate, travel companies, and political parties, his social influence is based primarily on the UCKG and its large number of worshippers and alliances with other evangelical and Pentecostal groups, which can influence local or even national elections in Brazil (Oro and Romera 2005, p. 13). Solely in Brazil, the UCKG is said to have 7 million members. This high number, however, provided by the church itself, is also adopted in scholarly works and consequently leads to mutual irritation. However, the stated number of worshippers seems to be much too high and requires some critical reflection. Although, the reference to the high membership numbers of Millions of people and its national and international growth rates fits perfectly with the self-portrayal of the UCKG, as well as on the academic side of the relevance justification to do research about the church. If one compares the high number of members provided by the UCKG with that of the Brazilian national census, a different picture of the UCKG emerges. The national census in Brazil determined the number of affiliates of the UCKG in Brazil to be less than 1.9 million in 2010. In 2000, however, the UCKG still had 2.1 million members, with a concurrent population increase of 11.05% from 2000 to 2010 in Brazil.^1^

The latest figures indicate that the number of members of the UCKG is in a sharp decline in Brazil, which could be linked to the national scandals the church and its bishops have caused and could justify the increased expansion abroad.

The international expansion of the UCKG can be dated to the year 1986 when Macedo himself emigrated to New York City (Mattos 2021, p. 401) and selectively assimilated religious models and discourses (McClymond 2021, p. 451). Since then the church mushroomed all over the world and claims nowadays to be active in 105 countries, having more than 8 million members worldwide. The UCKG has also spread to various countries in Europe (Jenkins 2007, p. 90; Oro 2019, p. 309). In Germany, the UCKG has been active since the 1990s and attracts members primarily from the migration milieu (van der Hoek 2021). Compared to cities like London and Paris, where the UCKG planted several congregations and conducts its services in prestigious buildings such as the Rainbow Theater in London, the UCKG in Germany has just eight locations throughout the whole country.

The headquarters of the German network is located in Berlin-Wedding, where the UCKG conducts its services in the New Nazareth Church. The church is conveniently situated in an area characterized by Turkish, African, and Arab migration. The Sunday services are attended by less than 100 people. Many other events and services that take place from Monday to Saturday are frequented by only 20 or 30 people.

Although the UCKG has been present in Berlin for 30 years and has not attracted negative attention in any way, it has a decidedly adverse standing in the local German press, which in 2019 even led to the local mayor wanting to oust the church from its current premises.^2^ The founder’s immense private fortune, national and international scandals involving other pastors and bishops of the UCKG, and various indictments and accusations guarantee the UCKG a high degree of visibility thru external media attention which was especially visible during the COVID-19 Pandemic (Martins and Miletta 2021).

## Doctrines, controversies, and epistemes about viruses and health inside the UCKG

The distinctive teachings of the UCKG are often reduced to the writings and sermons of its Bishop Macedo, who can be portrayed as a central charismatic leader of the worldwide network of the UCKG (Silveira Campos 1999, p. 366). In Macedo’s various publications, devils and demons are frequently described as the cause of evil, misfortune, and other problems in the history of humanity. All kinds of negativities, such as diseases, lovesickness, loneliness, and even physical as well as mental disabilities are directly or indirectly attributed to the presence and activities of Satan and his demons. Neither God nor even human beings can be the reason or the cause for any negativity because God created human beings perfectly and his will is for all people to enjoy physical and mental health, pure happiness, and financial prosperity (Macedo 1996, pp. 55, 1997a, pp. 90–91, 1997b, p. 60, 2019, p. 95). Consequently, illnesses must have been caused by demons whose goal is to destroy God’s perfect creation and blessings for human beings (Macedo 2019, p. 66, 96). Devils are therefore not only understood as pathogens in Macedo’s demonology but also bacteria and viruses are thus interpreted as the pure embodiment of demons (Ibid. p. 96). According to Macedo’s teachings, Demonic possession can be recognized by certain symptoms: Nervousness, severe and persistent headaches, sleep disturbances, phobias, frequent and sudden fainting spells, suicidal thoughts, visions, addictions, and depression are the most common signs that a person is possessed and oppressed by demons (Macedo 1999, pp. 63–64, 2019, pp. 65, 69–74). However, suffering from diseases does not necessarily mean being demonically possessed, but being possessed does subsequently mean suffering from a certain illness that can manifest itself in various ways and keep people from living a healthy and prosperous life according to God’s will (Macedo 1999, p. 64). In many cases, a crisis in an individual’s life is the cause of a person falling into the demons’ sphere of influence and consequently becoming possessed. Especially migration and tourism can lead to the transmission of demons through mere physical encounters with people in airports, queues, or mass events (Macedo 2019, pp. 43–44).

Macedo also uses social media platforms such as Facebook and Instagram, as well as the video platform YouTube, where he has a wide reach and a huge number of followers.

As I mentioned earlier, during the COVID-19 pandemic, the UCKG and the specific teachings of Macedo have been addressed in numerous studies. In an earlier survey, Fernando and Martins analyzed the changing rhetoric of Macedo since the spread of the COVID-19 virus over a 90-day period, analyzing his social media content. In the initial phase, Fernando and Martins found an overlap with the statements of the Brazilian federal government, which assumed a global conspiracy and misinformation (Martins and Fernando 2021, p. 42). However, as the pandemic unfolded, Macedo found the strategy to make more sense to acknowledge the reality of the virus and change its rhetoric to one of demonic siege by exaggerating the danger posed by the virus through demonization (Ibid. p. 42). Likewise, other studies have indicated that Macedo continued demonology in relation to the COVID-19 virus (Serrão and Chaves 2020, p. 246), insisting on keeping churches open as it would be worse for people to lose the protection of God than to be infected with the demonic virus (von Sinner and Zeferino 2022). Whether and to what extent Macedo wields interpretive authority over viruses and diseases in local churches abroad is what this article aims to explore. Recent global developments provide examples that Macedo’s influence and that of the UCKG’s Brazilian ruling clerical elite are far from as powerful as has often been implied. In Angola, for example, 330 UCKG pastors and bishops split from the Brazilian leadership in November 2019 (Sampaio 2020, p. 125), expressing their rejection of spiritual authority through the Manifesto Pastoral.^3^

## Methodology and research question

To address the research question of this article, the findings of the ethnographic field research conducted at the German headquarter of the UCKG in Berlin will be drawn upon. The research was originally conducted under the question, of how Brazilian Pentecostal churches in Berlin can be understood as self-organizations of migrants and which social resources and networks of solidarity the visitors provide for each other (van der Hoek 2021). The project was scheduled for the period from January to June 2020 and included next to the UCKG in Berlin also congregations from other Brazilian networks of Pentecostal churches such as Assembleia de Deus, Lagoinha, and Videira. To get access to the churches and to meet their visitors, I regularly attended services from October 2019 and conducted detailed observation protocols. However, one aspect that was not considered in the planning and initial implementation of the project was the possibility that a global pandemic and a novel virus would arise during the time of participant observations and data collection, which could fundamentally change the discourse and practices on viruses, health, and demons within the local UCKG in Berlin. Through the detailed observation protocols, results can be analyzed retrospectively under a modified research question. This question would be, how the local church of the UCKG in Berlin adopted its discourse on demonology before and during the emergence of the COVID-19 crisis and how the UCKG reacted and responded to the regulatory restriction measures of the German government. In total, 17 services were observed and logged during this time. It should be noted in advance, that in the period between March 21 and June 15, a strict lockdown of almost three-month prevented the UCKG from gathering and made the participant observations during this period impossible.

In order to classify the context and the overall social and political reactions as well as the pandemic developments from the respective periods, the releases of the federal press conferences and political resolutions were retrospectively evaluated in a documentary analysis and placed in context.

The following empirical section of this article can be divided into four parts, each covering four different time periods. The first section describes initial observations from November 2019 to January 2020. This is the time when the COVID-19 virus was discovered and started to spread out from Wuhan to other regions. The second section describes the occurrence of the first COVID-19 documented infection in Germany on January 27 and the reactions of the UCKG. The third section covers the period of the first infections in Berlin, when government actions led up to the first lockdown in Berlin on March 21. The fourth section covers the immediate UCKG reactions and responses in Berlin in the period after the first lockdown. The final section analyzes the observations and relates them to the concept of *Third Space *of Homi Bhabha.

## Observations from November 2019 to January 2020

The very first visit to the UCKG in Berlin was on November 1, 2019. It was a deliverance service, on a Friday evening. The small group of 20 worshippers waited devoutly sitting on wooden chairs in a small room just before the service began. Interactions between the visitors hardly took place. Occasionally, some of the visitors greeted each other, while others stared blankly at the altar or prayed silently. The services on Friday are meant for the deliverance of demons and demonic powers worldwide. The day of the week is not arbitrarily chosen. It corresponds to the day of the week on which services of Afro-Brazilian cults, such as Umbanda and Candomblé are conducted and is thus understood as a spiritual counterprogram (Oosterbaan 2017, p. 46). The UCKG incorporates the demons and spirits of Umbanda and Candomblé into their cosmology and spiritual practices, which is obviously also adopted in the churches outside of Brazil. In addition, services are offered in Berlin every single day of the week, up to three times per day. Along with Sunday services, Friday services are one of the most widely attended services in the UCKG. This corresponds with the results of a previous survey that was conducted by other researchers, who found through interviews at the Berlin location of the UCKG, that the migrant visitors in particular greatly appreciate the services offered, since the UCKG in Berlin conveys clear ideas about what is right and what is wrong, and thus provides orientation (Rodrigues and Silva 2014, p. 107). The services include songs, prayers, and sermons. From the first observations, it was noticeable that special importance was given to the sacral place of the church building and its artifacts. The presence of God at the altar is interpreted by the adherents as being particularly strong and prayers in the physical proximity of the altar are regarded as very effective. This can be seen, for example, in the observation that adherents would go to the immediate vicinity of the altar before the service starts, to speak a prayer in the very presence of God. Also, during the services, visitors are repeatedly asked by the pastors to come forward to speak prayers or receive specific blessings nearby the altar. The adherents then stand close together, speaking in tongues or reciting prayer formulas spelled word by word by the pastor or one of its assistants. There is usually close physical contact between worshippers. Physical contact occurs in situations for example, in which the pastors or their assistants place their hands on the heads of the adherents and pray for them. Prayers are used to expel demons and negativity from the physical body of the adherents. In doing so, the adherents stand close to each other and speak loudly and firmly to address the demons. In some cases, the assistants and pastors get very close to the worshipper and speak closely into their ears. Especially during exorcisms, intensive physical contact takes place. Oosterbaan describes exorcisms as central rituals in the UCKG, that help the adherents to address problems in their life which are interpreted spiritually (Oosterbaan 2017, p. 11). Adherents are thus given the opportunity to verbalize concerns and fears in various ways during the service. Believers also communicate problems regarding their employment contracts, health conditions, and visa arrangements through exorcisms. However, exorcisms occur quite seldom and experiences with demons are distributed more often virtually on TV among adherents. Just like Edir Macedo, also the UCKG and the German pastors are active on YouTube, where they post videos of themselves or other adherents talking about their personal experiences that they made with God and with other supernatural beings, such as Demons.

During my visits, only once happened an exorcism at the very beginning of the research project. In the church service when a demon manifested itself and an exorcism took place, one of the people present suddenly lay on the floor while being prayed for. Two of the assistants bent over the person and ordered the demons to disappear with outstretched hands. The person rolled and visibly resisted the prayer and the actions of the assistants. The pastor attempted to calm the audience and asked them not to pay attention to the exorcism. When the pastor realized that the assistants were not successful in calming the person on the floor and the exorcisms, were not effective, the pastor personally intervened, grabbed the person by his jacket, and ordered the demon that possessed the person strictly to manifest itself in the name of Jesus. The person then spoke slowly in a deep and somber voice, saying that the demons that possessed the person were millions and responsible for depression and other problems in the person’s life. The pastor finally succeeded in expelling the demons, after he got all the information he needed and the person went visibly thankful and relieved back to his seat.

The sermon series at the very beginning of the observations dealt especially with physical and mental illnesses, which were thematized and interpreted based on the *Ten Plagues* from the book of Exodus Chapter 8. Each week, the pastors taught another plague and related them to the lifes of the believers. For example, the gnats and insects infesting the scalps of the Egyptians (Exodus 8:12) were interpreted by the pastor as inner doubts, fears, and headaches that cause the believer to turn away from God and a will for man to live a good and healthy life.

Likewise, healing stories from the New and Old Testaments were regularly received to provide solutions, such as the story of when Jesus healed the blind man in the Gospel of John (20:31) or of Elisha and Naaman (2 Kings 5:8–19).

Being healed by casting out demons was a constant interpretation in the initial phase of the research project. Believers were taught by different pastors that doubts can get in the way of faith and keep them away from blessings and health. One of the pastors explained in one of his sermons that doctors and conventional medicine may provide quite powerful tools to defeat diseases. He, as a pastor, also seeks out doctors when he has a physical ailment. Nevertheless, other visitors to the events repeatedly testified to having experienced healing miracles in the UCKG. No major healing miracles took place during my observations and were therefore not documented. However, miraculous healings in Berlin were often related by the audience to invisible ailments, such as abdominal pain, fatigue, and headaches. In addition, videos were regularly presented by the pastors which portrayed adherence from other parishes of the UCKG who reported in their testimonies that they were possessed by demons that caused severe illnesses and described the diseases and symptoms in detail, to talk about the cure by the Holy Spirit or spiritual objects provided by the UCKG. The illnesses were often fatal diseases for which the doctors knew no more advice and therapies brought no cure. Through the sanctified water from the church or through faith or through prayers or donations to the church, the people were miraculously healed.

The theme of demons was also raised on cultural occasions and interpretations on specific dates. Worshippers were especially warned about Friday the 13th of December 2019 and asked if they could, by all means, attend the deliverance service and bring personal clothes and textiles of those relatives who could not come, so that the clothes could be impregnated with spiritual protection against any negativity and especially diseases. The reason for these measures is that an above-average number of misfortunes happen on Friday the 13th, as demons are particularly active on this day, the pastor interpreted. Some of the visitors also brought photos of relatives, so that those pictured would benefit from the blessings and experience good health. In some services, even family photo albums were held up while the pastor prayed so that the blessings would pass to everyone inside that photo album. In the context of healing and health prevention, worshippers were given small medicinal vials. These vials contained a mixture of water, olive oil, and red grape juice with which the visitors were asked to disinfect their hands. The visitors also were advised to take the vials home and rub the contents of the vial on their hands while washing their entire bodies. Healing from the consecrated water would be effective against any disease. The believers were warned by the pastors not to let their surroundings defile them. They are constantly in danger of ‘defiling’ themselves.

In the context of the Old Testament, one of the sermons addressed the issue of generational curses (Exodus 20:5; 34:7; 4). As such, the pastor also interpreted addictions and genetic predispositions that can be passed on within families and lead to deteriorated health or premature death. Diabetes or cardiovascular diseases were therefore interpreted as a generational curse. The pastors actively prayed with the congregation for deliverance from such curses. Likewise, reference was made to the preparation of a prayer campaign in which the pastors of the UCKG will collect letters with written intercessions from the adherents and bring them to Mount Sinai in Egypt to pray for their intentions there. The discourse of physical, mental, and spiritual health had a dominant influence on the themes of worship in the early days of the research and was described on several occasions. In the pastors’ sermons, this concern was repeatedly brought forward and translated into the life of the adherents with different biblical narratives and subsequent explanations.

## The arrival of the COVID-19-virus in Germany and the reactions and responses inside the local UCKG in Germany

On January 27, the first case of COVID-19 infection occurred in Starnberg, Bavaria. Four additional cases of COVID-19 infections were reported on the following day. The infected individuals were immediately isolated. Jens Spahn (CDU), the health minister of the federal republic of Germany at this time, was quoted as saying “It was to be expected that the virus would also reach Germany. But the case from Bavaria shows that we are well prepared for it. The risk to the health of people in Germany from the new respiratory disease from China remains low, according to the Robert Koch Institute.”^4^

On January 31, the pastor in the UCKG in Berlin continued his elaborations on the Old Testament story from 2 Kings 5 about the miraculous healing of Naaman by the Prophet Elisha. The pastor talks at length about ‘the fact’ that there are diseases for which classical medicine has no answer, yet a cure can never be ruled out. Mankind makes the paradoxical experience that despite ever-improving technology and medical progress, new diseases and viruses appear against which medicine has no answer. More allergies, novel bacteria, viruses, and pathogens are a symptom that Satan still dominates the earth, the pastor said. At no point did the pastor refer to the new appearance of the COVID-19 virus in Germany. Thus, the impression is created that the pastor’s statement is consistent with global events and that there is no need to explicitly mention the new virus. Faith is taught to the visitors as an adequate remedy against the devil and demons. The pastor use analogies between the Titanic and Noah’s Ark to portray that when God is on the side of believers, even the most primitive technology is at an advantage over sophisticated technology and science. To give emphasis to the pastor’s sermon, videos are shown again in which believers from other parishes of the UCKG in German cities testify about their healing experiences through the spiritual practices and sacred objects provided by the UCKG. The pastor shows a video of a young woman in the congregation in Frankfurt who was healed of a disease for which the doctors in several hospitals knew no answer. After the woman received the consecrated water from the UCKG and drank it, she was instantly healed.

On February 5, the health ministers from the European Union and the G7 states expressed their seriousness and desire to work more closely together to contain the global spread of the COVID-19 virus.

In the deliverance service of the UCKG in Berlin, above all, faith is propagated as the most powerful weapon in the fight against the devil and its domination of the world. The visitors were told by the pastors that there is an existential battle between the devil and the angels, which would decide about life or death. There will never be peace with the devil, the pastor said. The battle is aimed at the total destruction of the opponents. Here, for the first time during my observations, the pastor unfolds a detailed physiognomy of the demons, in accordance with Macedo’s descriptions (see above), characterizing them as spiritual beings that can enter the bones, joints, and cells of people with a physical body and must be exorcised from them with prayer. Ampoules with a mixture of water, grape juice, and olive oil were distributed. The believers were instructed to use the content of the ampoules and have them refilled as soon as possible. For the second filling, the adherents will receive a mixture of sanctified water and salt. The meaning behind this process was not explained to the visitors.

On several occasions, visitors were instructed to testify strongly to their faith and to appear confident in their daily situation. If they feel symptoms of illness or fatigue, they must not express this verbally under any circumstances because words create reality, according to the pastors. Those who suffer already from disease and say to themselves that they are sick would thus only aggravate their desolate conditions. Instead, it would cure them to express their own health and good condition with firm conviction, as this will have a positive effect on their body. Strengthening one’s faith would be in the foreground and a strong faith would provide a healthy physical and spiritual body. This theme became also visible in another sermon in this period, which addressed fake news and its spread. Getting the right information is important to be able to assess situations correctly, the pastor taught the visitors. The adherents were asked not to become unsettled by possible fake news. The pastor did not give a concrete definition of what fake news is and whether fake news would be spread in connection with the reports on the new COVID-19 virus. At the time, media coverage about the new Pandemic included footage from Bergamo, Italy, and was prominently discussed. Patients were being treated in the corridors of overcrowded hospitals or had to be flown out of the country as local healthcare services collapsed, which provided a surreal atmosphere at this time across Europe. In light of the dramatic developments in Italy, German Health Minister Jens Spahn feared on February 24, 2020, that the COVID-19 virus could spread further in Germany and cause serious problems to the public health service. “The situation in Italy also changes our assessment of the situation: corona has arrived as an epidemic in Europe […] Therefore, we must expect that it can also spread in Germany”^5^, the federal Health Minister said in a statement to the press. He added that Germany is prepared for this in the best possible way. In order to gather knowledge about the virus and improve therapies and vaccines, Spahn promised further funding for research and the development of a vaccine. On February 26, additional COVID-19 infections were confirmed in the most populous state of Germany, North Rhine-Westphalia, as well as Baden-Württemberg.

In the UCKG in Berlin, during the following services in February and March, medicinal vials containing a mixture of salt and water continued to be distributed wordlessly to the audience of the church services. Visitors were constantly advised for daily use to spread a little of this water on their heads and rub it in. the descriptions were as detailed as a hygienic prescription for disinfection, however, there was still no mention of the novel COVID-19 virus, nor that actions of the churches would provide effective protection.

In the phase of the first COVID-19 infections across Germany, the UCKG in Berlin has represented a discourse from the beginning by taking the danger of all viruses seriously and as a reality, even though COVID-19 was never mentioned by name. Unlike Edir Macedo in Brazil, who referred to the virus as harmless flu at this time (Martins and Miletta 2021, p. 38), the UCKG in Germany took all kinds of viruses even more seriously and tried to advise their flock within the scope of its cosmology and doctrines, without calling it by name. Through this discourse, which was different from that in Brazil, a *Third Space* opened up within the global network of the UCKG. From this dichotomy, it can be explained that the UCKG is in an incubational global moment that represents a common transition phase and is interpreted differently by the actors due to the different locations.

## First COVID-19 infection in Berlin until the lockdown

On March 1, 2020, the very first case of COVID-19 infection was confirmed in the city of Berlin. Only a few days later on March 3, the federal government banned the export of medical protective equipment (respirators, gloves, protective suits, etc.) and took further measures to contain and protect the population.^6^ One week later, 42 more people were infected in a local discotheque in Berlin. The mayor of Berlin consulted with a scientific board on measures to contain the virus. In the Senate Assembly, a ban on events that would gather more than 1000 people was discussed for the first time. On March 9, the Federal Minister of Health spoke at the Federal Press Conference on the current situation of the COVID-19 virus and pleaded for the social engagement of the German population.^7^ On March 11, 2020, in a press conference with then-Chancellor Angela Merkel (CDU), the head of the Robert Koch Institute, and the Health Minister called for a general ban against mass events with more than 1000 spectators.^8^

Regardless of the dramatic and hectic events in Germany, the discourse in the UCKG continued and the naming of the novel virus did still not take place. In the UCKG, the demonology of all kinds of diseases continued in a modified manner. The devil was described in a sermon in early March as omnipresent, seeing everything and being everywhere. Spiritual warfare is decided by the moral actions of a person, explained one of the pastors to the visitors. People outside the church are living in chaos and their lifes were marked by experiences of unhappiness, the pastor said.

Still, small medicinal vials continued to be distributed to the visitors during church services without any instructions on their actual meaning. Adherents continued to be encouraged to rub themselves with the water of the vials on a daily basis. The forms of prayer differed greatly compared to the worship practices at the very beginning of the observations. Exorcisms and intense prayer sessions involving physical contact were no longer practiced. Rather, during prayer, the worshippers were now asked to place their own hands on their heads and pray quietly for themselves. Although the pastors and assistants still avoided mentioning the COVID-19 Virus by name, measures against the COVID-19 virus were identified that changed practices of worship.

Particularly visible were the changes in the worship practice of the UCKG on Friday 13th of March 2020. In contrast to Friday 13th December 2019, the effectiveness of the worship practice was not addressed and the protection through the blessings was thematized. The service ended abruptly and early. Although it was a Friday the 13th, the pastor did not make a point of blessing garments, speaking long prayers in which demons were to be defeated and destroyed, or seeking close physical contact with visitors that day. Instead, the service wordlessly stopped 15 min earlier this day and no comments about demons and spiritual warfare were made.

On the evening of March 14, the Senate Chancellery of Berlin passes the *‘Verordnung zur Eindämmung des neuartigen Coronaviruses SARS-CoV‑2 in Berlin’* (engl. *‘Ordinance on the Containment of the new type of Coronavirus SARS-CoV‑2 in Berlin’*).^9^ All cultural institutions were ordered to close. Likewise, all sports fields, gyms, clubs, and bars had to be closed indefinitely. The decree was effective immediately. Public and non-public events and gatherings with more than 50 participants were not allowed to take place anymore (see §1).

The Sunday service in the UCKG on March 15, 2020, was conducted under the new ordinances. No more medicine bottles were distributed that day. A large jar has been placed in the center of the sanctuary and the visitors were invited to fill their personal bottles with consecrated water. For the very first time, visitors and adherents were asked to provide addresses and contact information before entering the church and to disinfect their hands with sanitizers. The chairs in the church were placed to the left and right at a distance of a minimum of one meter. According to the new regulations, there could now be no more than 50 people in the service. In fact, however, the 75 chairs provided were filled almost to capacity.

As usual, also the service just before the first lockdown started with a special prayer time for the sick, and the visitors were asked to drink the sacred water from the jar to stay protected from all diseases whatsoever. The collection of tithes, which were usually given by envelopes at the altar, were now collected by a cash machine, and adherents were asked to donate with their debit or credit card. The pastor assured the congregation that nobody needed to be afraid of becoming infected. Worse than the COVID-19 virus is the virus of unbelief and doubt for mankind.

On March 21, three weeks after the first COVID-19 infection case in Berlin became known, the health administration passes the decree for a curfew. The political situation changed daily, and dramatic pictures and footage from other European cities provided by German media shocked the population with dramatic scenes. Subsequently, the Senate of Berlin closed cafes and restaurants, and only pick-up of food and delivery services remained allowed. Likewise, all churches and other religious or non-religious places of assembly were closed. At that time, it was not apparent to the UCKG and other religious and cultural organizations whether the lockdown would be for a few weeks or several months.

## The UCKG in Berlin after the lockdown

The first church service that took place in the UCKG after the lockdown was held on June 15, 2020. For almost three months, the church in Berlin remained closed. The number of worshippers who gathered together was comparatively small. As usual, the service began with a prayer addressing demons as the cause of evil. However, evil underwent a discursive redefinition in the post-Lockdown period in the UCKG in Berlin.

Interestingly, the demons were now addressed exclusively as those that block financial success and must be cast out through prayers. Through biblical passages, the pastors convincingly explained to the visitors that it was the will of God for all creation to live in prosperity and good financial condition. Demons in people’s life, as the pastor further elaborated, try to block God’s will by causing people’s financial failure. Considering the Book of Judges (6:5), parallels are struck with the people of Israel from the time of Gideon to the people of the congregation in Berlin. Just as the Midianites invaded the land of the Israelites and like the locusts took away the goods and food of the Israelites, so too do demons encroach on people’s financial conditions. As a way out, true and strong faith would protect the visitors from demonic activities, the pastor explained. Strong faith is witnessed through donations and practices. Just as Gideon sacrificed a young bull to testify his true and undeniable faith and was able to deliver the people of Israel from its enemies, so too, the Pastor said, the adherents are called to donate financial resources to testify their faith.

During the period before the lockdown, believers were urged to be critical of media reports for fake news. The pastors explained to the visitors to continue to be cautious and that a lot of information spread one day can already be falsified on the next one. This unsettles doubts and fears; which believers should not be unsettled by, the pastor said. Regarding the COVID-19 virus, the lockdown, and ongoing contact restriction measures, no statements or positions whatsoever were taken during the following events after the lockdown.

In the following services, the UCKG pastors preached that divine or human rules are good for all people to protect them from destructive chaos. People should obey the law and not complain about the decisions of politicians. One of the pastors used the example of road traffic, which takes care to protect the people involved. He explained that people are primarily responsible for their own mistakes and must bear the consequences. There is a call not to complain, but to handle situations constructively. Many people would complain about the federal government and blame it for mistakes and problems, the Pastors said. Believers however should fight for a successful and good life.

The last observation contrasts with the UCKG’s own action just before the lockdowns. While informing that state commandments and laws are basically good for the life of individuals, the UCKG overrides the regulations by allowing more people than 50 into the service. Rules were renegotiated in the UCKG just before and after the lockdown and contradicted its own teaching.

## Conclusion

In the context of the global COVID-19 pandemic, the plurality of worldviews and contingency experiences in modern societies has brought to light different reactions and responses from a variety of actors. In retrospect, the events in the UCKG in Berlin can be described as follows: While the virus was first classified in 2019 and spread globally from China around the globe in a matter of months, the pandemic events evoked a sense of synchronicity regarding the measurements to fight the spread of the virus. Even if there were regional and national differences in how its effects were handled. Social distancing and sanitization were at that time the only effective courses of action used in almost all countries with the emergence of the virus. As in many other organizations, also inside the local headquarter of the UCKG in Germany, the central actors, namely the pastors were initially irritated by the crisis. These irritations became particularly visible in the UCKG, as it is a church with standardized processes and unified services around the world. However, transnational religious communities of the UCKG in Germany differed in its action and coping strategies from those of the UCKG in Brazil.

The different reactions and responses testify to a plurality, which is partially a result of migratory movements, missions, and transformation processes that forms hybrid spaces that create a *Third Space*. Prior to the pandemic, the topic of physical and mental health was addressed as a cross-cutting theme in all events at the UCKG of Berlin. During the period of observation, health and the connection with demonic possession became increasingly specific to physical pathogens and to the point of a detailed description in the physiognomy of demons until the first lockdown in March 2020. As an antidote, visitors were offered confidence in strong faith and medicinal vials of various sanctified liquids. In the context of global outreach, the rhetoric of the UCKG pastors in Berlin has changed over time.

The discursive shift in demonology, which had been particularly focused on health in the pre-Lockdown period, shifted radically to financial issues in the post-Lockdown period. This rhetorical shift allowed the UCKG to stay within its own doctrine and to switch in a flexible manner to a different aspect of its wide spectrum of demonology.

Although there was a broad front in Germany against the regulations of the federal government and the Senate of Berlin, which supported the refusal to wear medical masks in public buildings and transportation, it should be highlighted that the pastors of the UCKG pointed out that governmental rules are good for social coexistence and believers should not complain about state authorities.

The concept of *Third Space* helps to understand, that transnational religious communities are not static entities that can be characterized by a specific authenticity in each case. Although the UCKG and its global network are often collocated as a church that is organized as a centralized network and presents itself as such through uniform procedures, the findings of this survey demonstrate that there can be no blanket answer to how ‘the UCKG’ dealt with the COVID-19 crisis.

In the case of a global crisis, local social and political structures proved to be more dominant, than the global network and actors of the UCKG. Even though Edir Macedo continued to spread his interpretation of the Virus on his social media platforms and TV channel, the pastors of the UCKG in Berlin provided their response and reaction to the emergence of the novel virus. Discursively created spaces shift in the event of a crisis in the transnational context and open a new space that is detached from that of the home context. While before the pandemic the discourse endorses a causal connection between viruses and demonic possession, this traditional discourse space was radically abandoned and opened a new discourse of demonic possession and financial misfortune.

The *Third Space* can thus be understood not only as its physical location but as Bhabha presents, as a discursive space (Bhabha 1994, p. 216). For research on the Sociology of Religion, the synchronicity of events and the different reactions and responses to the COVID-19 pandemic provide a unique opportunity to engage in a nuanced way to research and analyze local congregations of transnational megachurches more carefully. The COVID-19 pandemic and the UCKG’s responses and reactions in Berlin provide a vivid example of how globally operating Pentecostal megachurches, often characterized by a narrow and centralized regime, can, in the face of a global pandemic, adopt or reject interpretive systems and switch into different modes of action.

## Footnotes

1 https://sidra.ibge.gov.br/tabela/137#resultado (accessed April 13, 2022).

2 https://www.dw.com/de/pfingstkirche-in-berlin-wedding-unerw%C3%BCnscht/a-50420019 (accessed October 13, 2022).

3 https://apublica.org/wp-content/uploads/2020/09/manifesto-pastoral6.pdf (accessed April 13, 2022).

4 https://www.bundesgesundheitsministerium.de/coronavirus/chronik-coronavirus.html (accessed April 13, 2022).

5 https://www.youtube.com/watch?v=Z5vUNVlARQg (accessed April 13, 2022).

6 https://www.bundesgesundheitsministerium.de/presse/pressemitteilungen/2020/1-quartal/weiterere-beschluesse-krisenstab-bmi-bmg.html (accessed April 13, 2022).

7 https://www.youtube.com/watch?v=H-OKJdtC8ro (accessed April 13, 2022).

8 https://www.youtube.com/watch?v=un7HjfSW8j4 (accessed April 13, 2022).

9 https://lexcorona.de/lib/exe/fetch.php?media=rechtsakteland:berlin:rechtsverordnung-corona-20200314-endg_fassung.pdf (accessed April 13, 2022).

## References

[CR1] Bhabha H (1994). The location of culture.

[CR2] Capponi G (2020). Overlapping values: religious and scientific conflicts during the COVID-19 crisis in Brazil. Social Anthropology.

[CR3] Chesnut AR (1997). Born again in Brazil. The pentecostal boom and the pathogens of poverty.

[CR4] Chesnut AR (2003). Competitive spirits. Latin america’s new religious economy.

[CR5] Fernandes S (2022). Christianity in Brazil. An introduction from a global perspective.

[CR6] Freston, Paul. 1993. *Protestantes e política no Brasil: da Constituinte ao impeachment*. PhD Thesis, Unicamp, Campinas.

[CR41] van der Hoek S (2021). Lusophony pentecostal churches in Berlin: religious identities between integration and transatlantic boundaries. International Journal of Latin American Religions.

[CR7] Jenkins P (2007). God’s continent. Christianity, islam, and Europe’s religious crisis.

[CR8] Koehrsen J (2016). Middle class pentecostalism in Argentina: inappropriate spirits.

[CR9] Kürbis, Ilka. 2021. Demografiebericht München – Teil 1. Analyse und Bevölkerungsprognose 2019 bis 2040 für die Landeshauptstadt. Landeshauptstadt München. Referat für Stadtplanung und Bauordnung. https://stadt.muenchen.de/dam/jcr:9f74fb22-9f40-49de-8c47-32110718ec9e/Demografiebericht_Teil1_2021.pdf. Accessed 24 Apr 2022.

[CR11] Leśniczak R (2022). News coverage of Christian churches and other religious bodies dealing with the Covid-19 pandemic: An analysis of newspapers in German and English. Church, Communication and Culture.

[CR12] Levin J (2020). The Faith community and the SARS-CoV-2 outbreak: part of the problem or part of the solution?. Journal of religion and health.

[CR13] Macedo E (1996). Vida com Abundância.

[CR14] Macedo E (1997). O Espirito Santo.

[CR15] Macedo E (1997). Plano de Poder: Deus, Os Cristaos e A Politica.

[CR16] Macedo E (1999). Doutrinas da Igreja Universal do Reino de Deus.

[CR17] Macedo E (2019). Orixás, Caboclos e Guias. Deuses ou Demônios?.

[CR18] Martins E, Fernando M (2021). A incorporação da pandemia na retórica da Igreja Universal do Reino de Deus. Calidoscópio.

[CR19] Mattos Ayres P, Wilkinson M (2021). Macedo, Edir. Brill’s encyclopedia of global pentecostalism.

[CR20] McClymond M, Wilkinson M (2021). Neo-pentecostalism. Brill’s encyclopedia of global pentecostalism.

[CR21] Ministério das Relações Exteriores. 2021. Comunidade brasileira no exterior—Estatísticas 2020. https://www.gov.br/mre/pt-br/assuntos/portal-consular/arquivos/ComunidadeBrasileira2020.pdf. Accessed 24 Apr 2022.

[CR22] Nachtwey O, Schäfer R, Frei N (2020). Politische Soziologie der Corona-Proteste.

[CR23] Oosterbaan M (2017). Transmitting the Spirit. Religious Conversion, Media, and Urban Violence in Brazil.

[CR24] Oosterbaan M, Linda van de Kamp JB (2020). Global trajectories of Brazilian religion.

[CR26] Openshaw K, Wilkinson M (2021). Universal church of the Kingdom of god. Brill’s encyclopedia of global pentecostalism.

[CR28] Oro AP, Ramos de Andrade S, Siuda-Ambroziak R, Stachowska E (2019). Considerations about Brazilian evangelical transnationalization to europe. Brazil Poland focus on religion.

[CR27] Oro, Ari Pedro, and Enrique J. Romera. 2005. The politics of the Universal Church and its consequences on religion and politics in Brazil. *Revista Brasileira de Ciências Sociais*. http://socialsciences.scielo.org/scielo.php?script=sci_arttext&pid=S0102-69092005000100005&lng=en&tlng=en. Accessed 27 Oct 2022.

[CR30] Robbins J (2007). Continuity thinking and the problem of Christian culture: belief, time, and the anthropology of christianity. Current Anthropology.

[CR32] Rocha C (2017). John of god. The globalization of Brazilian faith healing.

[CR33] Rocha C (2021). Global religious infrastructures: the Australian megachurch hillsong in Brazil. Social Compass.

[CR31] Rocha C, Vásquez M (2013). The diaspora of Brazilian religions.

[CR34] Rodrigues D, Silva M (2014). Imigração e pentecostalismo brasileiro na Europa: o caso da Igreja Universal do Reino de Deus. Revista Angolana de Sociologia.

[CR35] Sampaio CAM (2020). A Igreja Universal do Reino de Deus na “Reconstrução Nacional” de Angola. Religião & Sociedade.

[CR36] Serrão R, Chaves J (2020). Immigrant evangelicalism in the COVID-19 crisis: reactions and responses from Brazilian evangelical churches in Florida. International Journal of Latin American Religion.

[CR38] Silveira Campos L (1999). A Igreja universal do reino de Deus, um empreendimento religioso atual e seus modos de expansão (Brasil, África e Europa). *Lusotopie*. *Dynamiques religieuses en lusophonie contemporaine*.

[CR42] von Sinner R, Zeferino J (2022). Pandemic religion in Brazil—temptation and responsibility. Religions.

[CR39] Usarski F, Py F (2020). Religion and the pandemic—Latin American responses. International Journal of Latin American Religions.

[CR40] Vasconcelos de Castro Moreira L (2021). Self-othering in the testimonials of the Universal Church of the Kingdom of God in Madrid. Social Compass.

[CR10] de Vasconcelos L, Moreira C (2022). The secularisation of demons: exorcisms conducted by the Universal Church of the Kingdom of God in Madrid. Journal of Contemporary Religion.

[CR43] Yendell A, Hidalgo O, Hillenbrand C (2021). The role of religious actors in the COVID-19 pandemic: a theory-based empirical analysis with policy recommendations for action.

[CR44] Zurlo GA, Todd MJ, Crossing PF (2020). World Christianity and mission 2020: ongoing shift to the global south. International Bulletin of Mission Research.

